# The Influence of Gut Microbial Species on Diabetes Mellitus

**DOI:** 10.3390/ijms24098118

**Published:** 2023-05-01

**Authors:** Raghad Khalid AL-Ishaq, Samson Mathews Samuel, Dietrich Büsselberg

**Affiliations:** Department of Physiology and Biophysics, Weill Cornell Medicine-Qatar, Education City, Qatar Foundation, Doha 24144, Qatar

**Keywords:** *Bifidobacterium adolescentis*, *Bifidobacterium bifidum*, diabetes, gut, *Lactobacillus rhamnosus*, microbiome

## Abstract

Diabetes mellitus (DM) is a metabolic disorder with an alarming incidence rate and a considerable burden on the patient’s life and health care providers. An increase in blood glucose level and insulin resistance characterizes it. Internal and external factors such as urbanization, obesity, and genetic mutations could increase the risk of DM. Microbes in the gut influence overall health through immunity and nutrition. Recently, more studies have been conducted to evaluate and estimate the role of the gut microbiome in diabetes development, progression, and management. This review summarizes the current knowledge addressing three main bacterial species: *Bifidobacterium adolescentis*, *Bifidobacterium bifidum*, and *Lactobacillus rhamnosus* and their influence on diabetes and its underlying molecular mechanisms. Most studies illustrate that using those bacterial species positively reduces blood glucose levels and activates inflammatory markers. Additionally, we reported the relationship between those bacterial species and metformin, one of the commonly used antidiabetic drugs. Overall, more research is needed to understand the influence of the gut microbiome on the development of diabetes. Furthermore, more efforts are required to standardize the model used, concentration ranges, and interpretation tools to advance the field further.

## 1. Introduction

### 1.1. Diabetes Mellitus (DM) 

Diabetes mellitus is one of the main leading causes of morbidity and mortality worldwide [1]. It is a chronic metabolic disease characterized by hyperglycemia, an elevation in the blood glucose level caused by a defect in insulin secretion and/or action [2,3,4]. Diabetes is classified into three main types based on its genetics, etiology, and diagnostic criteria: type 1, type 2, and gestational diabetes [5]. Their complications in several organs, such as the heart, eyes, and kidneys, profoundly affect the patient’s quality of life [6]. Depending on the kind and duration of diabetes, the symptoms may include polyuria, polyphagia, polydipsia, and weight loss [7]. Currently, oral and injectable antidiabetic drugs, insulin therapy, and lifestyle management are the primary therapeutic modalities used to treat diabetes. However, the alarmingly high rate of diabetes worldwide shows the necessity to develop new and more effective therapeutic approaches to target the disease and its complications [8].

### 1.2. Gut Microbiome and Diabetes 

The human gut microbiome comprises 100 trillion bacterial species in the intestinal tract [9]. It is regulated by internal and external factors such as genetics, diet, and medications [10]. The gut microbiome influences the overall health status of an individual through nutrition, physiology, and immunity [11]. Disruption in the diversity of the gut microbiome is linked to multiple pathological conditions, including diabetes [12,13]. Gut dysbiosis and increased gut permeability result in the translocation of lipopolysaccharide, which can activate the innate immune system [14]. In diabetic patients, the level of lipopolysaccharide in the plasma was higher compared with healthy participants, resulting in low-grade inflammation that may have caused insulin resistance [15,16]. The observed inflammatory responses in diabetic patients may be caused by gut microbiome dysbiosis and their major metabolites, such as bile and short-chain fatty acids, which regulate glucose metabolism and insulin sensitivity [17]. This shows that the gut microbiome may be an essential driver of the pathogenesis of diabetes and can be used as a potential therapeutic target.

### 1.3. Gut Microbial Profile in Diabetes 

Two prominent phyla, *Firmicutes* and *Bacteroides,* are present in the gut, representing 60–80% of the species [18]. Changes in their abundance have been linked to multiple pathological changes [19,20]. In a study of 36 male participants, 18 of which were diabetic, the level of *Firmicutes* was significantly higher in the control group compared to the diabetic group (*p*-value = 0.03) [21,22]. This suggests a possible positive correlation between diabetes and gut microbiome composition. Furthermore, the reduced level of butyrate-producing bacteria such as *clostridiales* sp. influences insulin sensitivity, low-grade inflammatory response, and glucose and fat metabolism in diabetic patients [23,24,25]. Not only at a phylum level but some bacterial species, such as *Lactobacillus*, have been linked to diabetes as they positively correlate with fasting blood glucose and glycosylated hemoglobin [26]. Taken together, more efforts are required as different cohort studies showed inconsistent findings.

The gut–brain axis in diabetes management has gained more attention recently as it might provide promising potential. The gut microbiome critically influences the glucose homeostasis pathway by interacting with energy-regulating centers in the brain about incoming nutrient materials [27]. This supports the importance of the metabolites produced by the gut microbiome.

Throughout the literature, the role of the gut microbiome in diabetes management is discussed. Here we evaluate and analyze published studies that report the influence of three bacterial species, *Bifidobacterium adolescentis*, *Bifidobacterium bifidum,* and *Lactobacillus rhamnosus,* on diabetes mellitus. Furthermore, we assess the impact of their effect on specific pathways. Finally, we identify gaps in the current research.

## 2. Search Strategy and Selection Criteria

Medline, Scopus, and PubMed were searched for manuscripts published from 2000 to 2023 using the search terms “diabetes”, “microbiota”, “microbiota AND diabetes”, “microbiome profile AND diabetes”, “gut microbiota enzymes”, “*Bifidobacterium adolescentis* AND diabetes”, “*Bifidobacterium bifidum* AND diabetes”, “*Lactobacillus rhamnosus* AND diabetes”. We selected eighty-five articles and analyzed them in detail for this review. Eligible studies included in vivo, in vitro, and clinical trial publications addressing the beneficial effects of selected bacteria on diabetes and its complications.

## 3. Diabetes Management Using Microbial Species

The development of diabetes is associated with profound gut dysbiosis [28]. Restoring the balance of gut microbiome composition by administering probiotics (live non-pathogenic microorganisms) in an adequate concentration has been reported to improve diabetes [29]. Several human and non-human studies reported the influence of using probiotics for diabetes. For example, in diabetic patients given yogurt containing *L. acidophilus La5* and *B. lactis Bb12* as probiotics, fasting blood glucose, insulin, insulin resistance, and glycosylated hemoglobin levels were reduced [30]. A meta-analysis of 520 type 2 diabetic patients reported that probiotic administration improved glycemic control and lipid metabolism [31]. Probiotic administration also influences oxidative status and inflammatory parameters in diabetic patients [32]. Seventy participants with diabetes were given probiotics for a month which significantly reduced the levels of IL-6, IL-1, IL-8, and TNF-a compared to the control group [33]. The data showed how probiotic administration influenced the inflammatory response in participants with diabetes. The administration of *Lactobacillus* for two months reduces uric nitrogen in the blood [34]. Additionally, probiotic administration influenced and regulated the level of glycated hemoglobin, total cholesterol, triglycerides, and low-density lipoprotein cholesterol in pre-clinical diabetes [35]. On the other hand, similar results were observed when the probiotic was given to animal models. The administration of *Bifidobacterium* for one month and *Lactobacillus* for three months in a mouse model with type 2 diabetes was reported to normalize glucose metabolism and insulin sensitivity [36,37]. One of the concerns with probiotic treatment is safety and tolerability. All the mentioned studies reported no adverse effects and probiotic usage was safe. Despite that, more efforts are required to standardize the protocol and estimate the proper dosage.

## 4. The Influence of Specific Microbial Species on Diabetes

**Figure 1:**
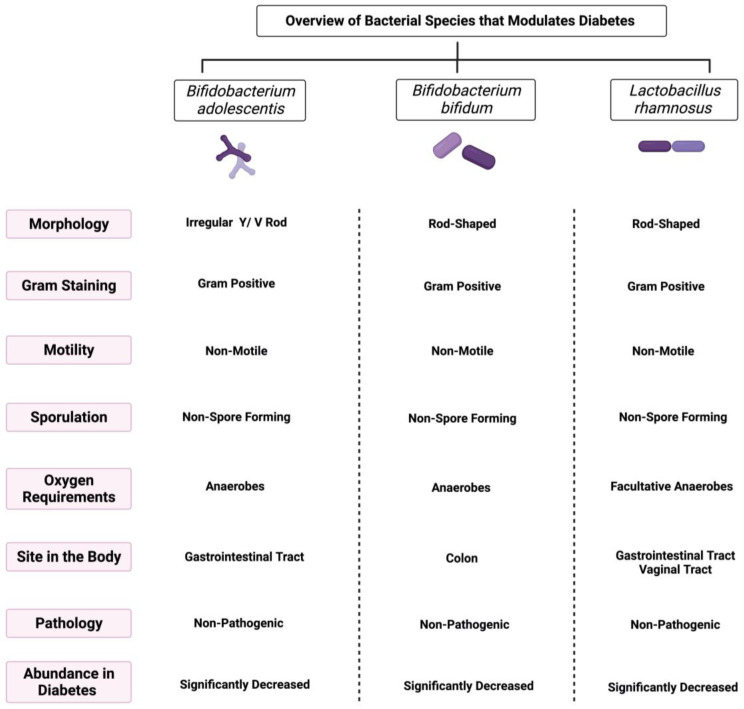
Overview illustration of bacterial species that modulate diabetes. The figure shows the main morphological and biochemical features of each species. Created with BioRender.com.

Throughout our research, multiple reports discuss the influence of three bacterial species, *Bifidobacterium adolescentis*, *Bifidobacterium bifidum*, and *Lactobacillus rhamnosus*, on diabetes mellitus. Here, we discuss each of them in detail and provide insight into the mechanisms by which they improve diabetes. Figure 1 highlights an overview of the three species and their main characteristics.

### 4.1. Bifidobacterium adolescentis

Bifidobacteria are Gram-positive, non-spore-forming, and non-motile bacteria known to be the first colonizer of the infant gut [38]. Their presence in the gut has been linked to several beneficial effects on the host as they prevent intestinal inflammation, colonic adenomas, and cancer [39]. *Bifidobacterium adolescentis* is a vital gut flora in adults [40,41]. In patients with type 2 diabetes, the abundance of *B. adolescentis* in the intestine is significantly reduced [42]. Using *B. adolescentis* (1 × 10^8^ cfu/mL) daily on twenty volunteers aged 50 to 60 for thirty days as a supplementation alleviates gut microbiome disorder and reduces blood glucose [43].

**Figure 2:**
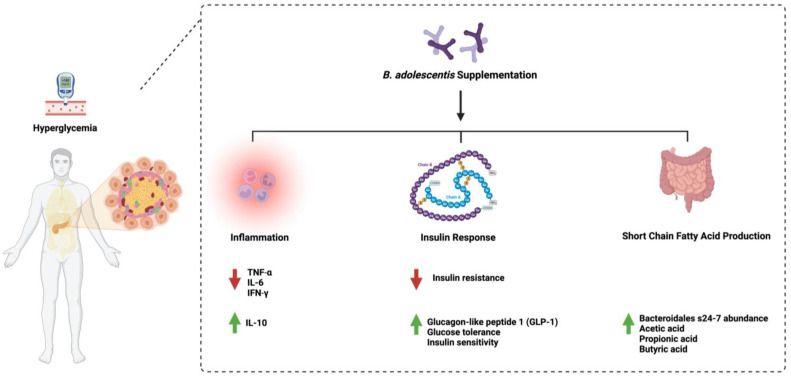
Overview illustration of *Bifidobacterium adolescentis* on diabetes. The figure shows the three main pathways: inflammation, insulin response, and the production of short-chain fatty acids by microorganisms. Created with BioRender.com.

Additionally, administering eight strains of *B. adolescentis* (2 × 10^8^ cfu/mL) for 12 weeks in diabetic mice restored gut microbiome homeostasis, alleviated inflammation, and increased the abundance of short-chain fatty acid-producing microorganisms [44]. Moreover, supplementing *B. adolescentis* (5 × 10^8^ cfu/mL) in mice fed a high-fat diet daily for twelve weeks improved insulin sensitivity and reduced visceral fat accumulation [45]. Unfortunately, the literature lacks more data that support or challenge the observed beneficial effects of *B. adolescentis* administration in diabetes. Furthermore, protocol standardization is required to ensure the safety and efficacy of using such an approach. Figure 2 highlights the main pathways affected by *B. adolescentis* administration in diabetes.

### 4.2. Bifidobacterium bifidum

**Figure 3:**
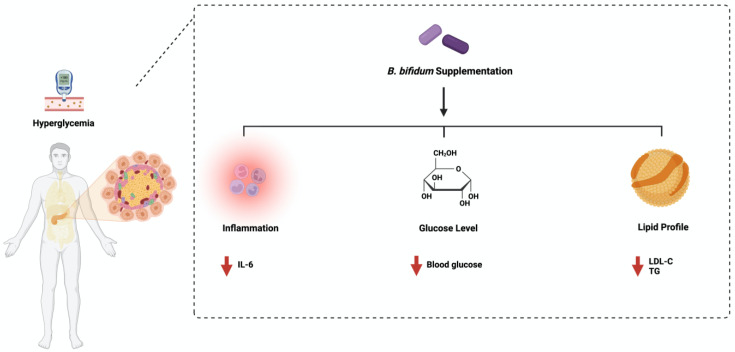
Overview illustration of *Bifidobacterium bifidum* on diabetes. The figure shows the three main pathways: inflammation, insulin response, and lipid profile. Created with BioRender.com.

*Bifidobacterium bifidum* is one species of naturally occurring microbiota detected in breastfed infants [46]. It is considered a dominant resident of the gut population [47]. *B*. *bifidum* consists of 3000 genes that encode carbohydrate enzymes such as glycosyl transferases (GTs), glycosyl hydrolases (GHs), and carbohydrate esterases (CEs) [48]. This showed the ability of *B*. *bifidum* to metabolize host-derived glycans such as human milk oligosaccharides and mucin [49]. Using *B*. *bifidum* in diabetes management has started to gain more scientific attention recently. A single administration dosage of 1 × 10^7^ cfu/mL daily for 28 days reduced fasting blood glucose, glycosylated hemoglobin, triglycerides (TG), and total cholesterol in Wistar rats [50]. Additionally, diabetic patients treated with a collection of probiotics, including *B*. *bifidum* (2 × 10^9^ cfu/mL) daily for 12 weeks, significantly decreased insulin resistance, fasting blood glucose, and increased insulin sensitivity and HDL cholesterol level. It also improved the total antioxidant capacity and reduced the C-reactive protein level [51]. The combination treatment of different *Bifidobacterium* spp., including *B*. *bifidum* and excluding *B. adolescentis,* ameliorated insulin resistance and reduced blood glucose levels in mice [52]. More studies are needed to evaluate how *B*. *bifidum* manages diabetes. Additionally, studies that address the influence of *B*. *bifidum* and *B. adolescentis* may be essential for better treatment outcomes. Figure 3 highlights the main pathways affected by *B*. *bifidum* administration in diabetes.

### 4.3. Lactobacillus rhamnosus

*Lactobacillus rhamnosus* was first isolated in 1983 and is known for its ability to resist stomach acidity and strong avidity for intestinal cells. It has been widely used in targeting multiple pathological conditions, such as cancer, as an effective probiotic [53]. Administering *L. rhamnosus* daily (1 × 10^8^ cfu/mL) in rodents for four weeks improved glucose tolerance by reducing endoplasmic reticulum stress [54]. Additionally, in mice fed a high-fat diet, treating 10^9^ cfu/mL of *L. rhamnosus* daily significantly reduced the insulin level and fasting blood glucose. It also reduced proinflammatory cytokines such as IL-6 and TNF-a [55].

**Figure 4:**
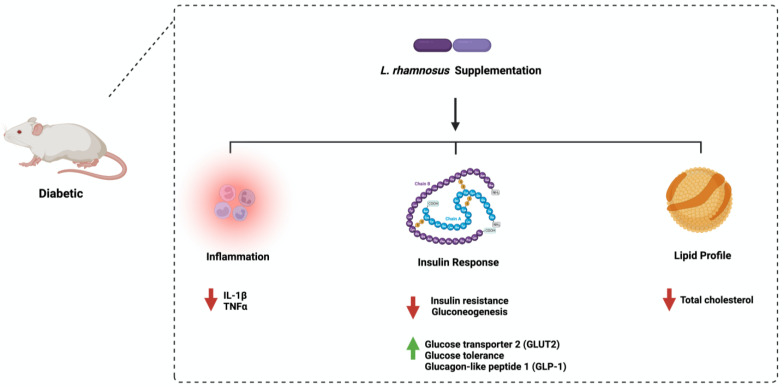
Overview illustration of *Lactobacillus rhamnosus* on diabetes. The figure shows the three main pathways: inflammation, insulin response, and lipid profile. Created with BioRender.com.

Furthermore, oral administration of *L. rhamnosus* improved glucose tolerance in diabetic rats by downregulating the expression of glucose 6 phosphatase [56]. The administration of *L. rhamnosus* to diabetic mice reduced insulin, glycosylated hemoglobin, and fasting blood glucose levels and increased glucagon-like peptide 1 levels in serum [57]. Similar results were obtained when 3 month old male Zebrafish were used [58]. These observations show the urgent need for protocol standardization and model specification to estimate the beneficial effect of *L. rhamnosus* in diabetes. Figure 4 highlights the main pathways affected by *L. rhamnosus* administration in diabetes. Table 1 summarizes the data available in the literature that address the influence of the species *Bifidobacterium adolescentis*, *Bifidobacterium bifidum*, and *Lactobacillus rhamnosus* on diabetes mellitus. The table includes essential data about the targeted pathway tested, the mode of administration, the effects on diabetes, the follow-up period, and the method and model used in each study.

## 5. Discussion

Diabetes is a global metabolic condition with a high incidence rate worldwide. Developing new and improved therapeutic approaches to target the disease and its complications is necessary. The gut microbiota has been linked recently to diabetes. Here, we searched the literature and reported the role played by the three commonly addressed microbial species on diabetes: *Bifidobacterium adolescentis*, *Bifidobacterium bifidum*, and *Lactobacillus rhamnosus*. Both animal and human studies reported the influence of *Bifidobacterium adolescentis* administration on blood glucose level, an abundance of short-chain fatty acids, and inflammatory response in a dose dependent manner that ranges from 1 × 10^8^ to 5 × 10^8^ CFU/mL. Moreover, administering *Bifidobacterium bifidum* (1 × 10^7^–2 × 10^9^ CFU/mL) reduced fasting blood glucose, insulin resistance, and improved sensitivity in human participants with diabetes and animal models. Unfortunately, this is not the case with *Lactobacillus rhamnosus* as most of the available studies reported the role of this species on diabetes in animal models only. Despite that, the data support the positive influence of this species on insulin resistance and lipid profile.

Throughout the literature, we observed the lack of standardization regarding the protocol followed, the model used, the diet used to induce diabetes in animal models, and the mode of administration, as most studies followed oral or intraperitoneal administration. Establishing standardized protocols that specify specific guidelines will help further advance the field. Additionally, the literature shows that many studies investigate a single microbial species. The gut microbiome is a community of microorganisms interacting with each other and the host. Isolating and investigating a single organism only might not be of great interest. As a starting point, a study may investigate the influence of the three bacterial species mentioned in this paper on diabetes in human and animal models and report the challenges and limitations. By doing so, we can then, step by step, look at the gut microbiome as a community in the context of health and diseases. The following sections highlight some essential topics that need further discussion and research for better treatment outcomes.

### 5.1. The Influence of Combination Therapy on Diabetes 

Diabetes is managed mainly by antidiabetic drugs such as metformin [61]. Its administration augments glucose uptake in tissues and reduces glucose output [62]. Due to its high efficacy and safety level, metformin is used as the first line of treatment in patients with type 2 diabetes [63]. Various research supports the influence of metformin on the gut microbiome [64]. In a randomized study of patients with type 2 diabetes, the administration of metformin altered the composition and function of the gut microbiome. The results also showed how metformin prompted the growth of *B. adolescentis,* which was associated with reduced blood glucose levels [65].

**Figure 5:**
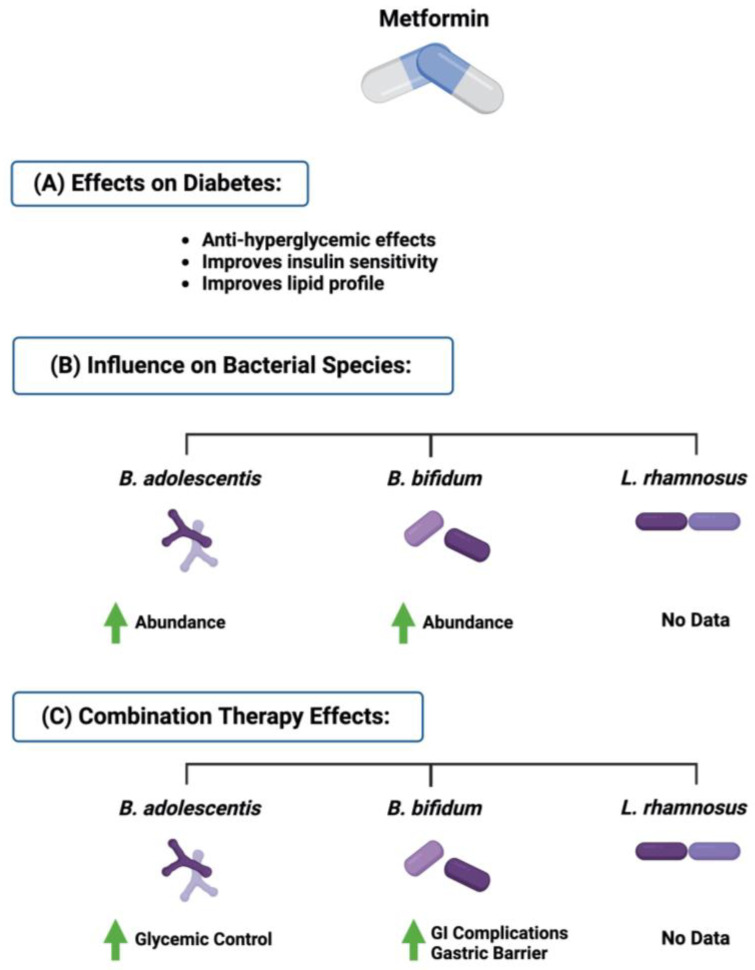
Overview illustration of metformin effect on the three gut microbial species discussed and the influence of combination therapy on diabetes outcome. Created with BioRender.com.

Additionally, metformin treatment altered the composition of the gut microbiome by enhancing the growth of *Lactobacillus*, *Bifidobacterium*, and *Escherichia* and reducing the abundance of *Intestinibacter bartlettii* [66]. Those reports prompted more research in the field of combination therapy and diabetes. The co-administration of metformin and *B. bifidum* in rats suppressed the metformin effect on feces while maintaining the antihyperglycemic effect of metformin [67]. Furthermore, the combination treatment of metformin and *B. bifidum* in 40 patients with diabetes for ten weeks significantly improved the gastrointestinal symptoms associated with metformin without altering the glucose control effect of the medication [68]. More studies are required to assess and evaluate those results on other bacterial species, such as *B. adolescentis* and *L. rhamnosus.* More research is needed to evaluate this approach with other antidiabetic drugs, such as sulfonylureas and meglitinides. Figure 5 highlights the influence of metformin on the three discussed bacterial species.

### 5.2. The Influence of Flavonoids on Species Abundance 

Flavonoids are natural compounds present abundantly in fruits and vegetables and exert several biological benefits, such as anticancer and anti-inflammatory properties. Our previous work extensively covers the influence of flavonoids and phytochemicals consumption on metabolic conditions such as diabetes and cancer. We also reported the relationship between the gut microbiome and flavonoid metabolism in the context of health and disease [69,70,71,72,73]. In this section, we report the influence of flavonoid consumption on the abundance of *Bifidobacterium adolescentis*, *Bifidobacterium bifidum*, and *Lactobacillus rhamnosus.* An in vitro stimulated fermentation method was used to evaluate the influence of nine flavonoids—hesperidin, hesperetin-7-O-glucoside, hesperetin, naringin, prunin, naringenin, rutin, isoquercitrin, and quercetin—in 10 healthy Chinese volunteers. The results showed that the administration of hesperetin-7-O-glucoside, prunin, and isoquercitrin significantly enhanced the abundance of *Bifidobacterium* spp. [74]. Additionally, adding quercetin significantly increased the abundance of *Bifidobacterium adolescentis* in particular [75]. Interestingly, and in another report, the administration of quercetin enhanced the quantity of *Lactobacillus rhamnosus* while inhibiting the growth of pathogenic bacteria [76,77]. This may support the synergistic effect of the same flavonoids on the abundance of different bacterial species in the gut. Research that supports those findings in the context of diabetes is lacking in the literature. We think conducting more research in that area can provide insight into a potential new treatment/management for diabetes. Moreover, studies that evaluate the efficacy and safety of using flavonoids in combination with other antidiabetic drugs are necessary. Furthermore, the bioavailability challenge accompanying flavonoid administration may be improved if we better understand the role of the gut microbiome.

### 5.3. Fecal Microbiota Transplantation and Diabetes

Fecal microbiota transplantation (FMT) transfers the stool sample of a healthy participant into the colon of a patient suffering from a medical condition to restore the typical abundance and function of the gut microbiota [78,79]. The procedure is considered well-tolerated and safe, with minor side effects such as abdominal cramps and diarrhea [80]. FMT has been used to treat metabolic syndrome, inflammatory bowel disease, and diabetes [81,82]. A 24-year-old patient with type 1 diabetes treated with FMT showed a graduate improvement in blood glucose level, glycosylated hemoglobin, and nutritional status. Additionally, the abundance of gut bacterial species changed after the treatment [83]. Furthermore, an open-labeled controlled trial of 13 patients with type 2 diabetes revealed that the treatment with FMT improved blood glucose levels, glycosylated hemoglobin, and the abundance of *Bifidobacterium* [84]. Furthermore, mice with type 2 diabetes were treated with FMT and reported an improvement in the level of insulin resistance while the level of inflammatory response was reduced. Additionally, Western blots and flow cytometry results reported inhibition of the apoptotic pathway after the FMT treatment [85]. Although none of the studies reported adverse side effects of the FMT treatment, more studies are required to assess and standardize the mode of administration, the concentration, and the safety of the procedure. Unfortunately, the literature still lacks more information that links the effect of FMT on restoring the abundance of the three bacterial species—*Bifidobacterium adolescentis*, *Bifidobacterium bifidum*, and *Lactobacillus rhamnosus*—in diabetic patients/models. Despite that, reporting the available data that support the positive influence of FMT on diabetes and gut microbiome profile, in general, may provide a roadmap for structured research in linking FMT to the three bacterial species and diabetes.

## 6. Conclusions

Diabetes mellitus is a chronic condition with a massive burden on patients worldwide. Developing new targets and management plans which can be used with the currently used treatment is essential. The gut microbiome has been recently used in diabetic research. Throughout our study, we observed a lack in the literature of data that addresses specific bacterial species and their correlation with diabetes. *Bifidobacterium adolescentis*, *Bifidobacterium bifidum*, and *Lactobacillus rhamnosus* are the most commonly addressed bacterial species with diabetes in the literature. Those bacterial species were reported to reduce the biochemical parameters of diabetes and improve its complications.

Unfortunately, the field still lacks standardization in the protocol followed, the models used, and the interpretations. Furthermore, more efforts are required to address the available online Atlases that discuss gut microbiome causality without solid evidence. Generally, the gut microbiome field will be essential in futuristic treatments, primarily when combined with other therapeutic options. However, more research is needed to evaluate the safety and efficacy of this proposed approach.

## Figures and Tables

**Table 1:** Representative bacterial species and their underlaying antidiabetic effects.

Bacterial Species	Targeted Metabolites/Proteins/Genes/Pathway	Mode of Administration	Number of Strain Tested/Concentration	Dietary Intervention	Follow-Up Period	Mechanism of Action	Methods of Testing	Model Used		References
								In Vivo	In Vitro	
*Bifidobacterium adolescentis*	Glucose Lipid metabolism Inflammatory markers	Oral gavage Intraperitoneally	8	High-fat diet	12 weeks	-Alleviate insulin resistance -Restore gut microbiota homeostasis -Increase the abundance of SCFA-producing flora -Alleviate inflammation by reducing the concentration of TNF-a, IL-6, and IFN-Y	Biochemical analysis Histopathological analysis SCFA analysis Polymerase chain reaction	-C57BL/6J mice	-Pancreatic cells -Hepatic cells	[44]
	Visceral fat accumulation Insulin sensitivity	Orally	5 × 10^8^ colony-forming units/mL of live *B. adolescentis*	High-fat diet	12 weeks	-Supplementation of this bacteria improved diabetes and insulin sensitivity by increasing the production of glucagon-like peptide 1 (GLP-1) -A reduced visceral fat accumulation (liver steatosis and mesenteric fat)	Insulin sensitivity Quantitative reverse transcription PCR Histological analysis	-Male Wistar rats	-Hepatic cells	[45]
*Bifidobacterium* *bifidum*	Glucose Lipid metabolism Inflammatory markers	Orally	1 × 10^7^ colony-forming units/mL	NA	28 days	-Administration of *B. bifidum* significantly reduced serum fasting blood glucose -It reduced the level of total cholesterol, triglycerides, low-density lipoproteins, and very low-density lipoproteins and enhanced the level of high-density lipoproteins -Reduced the activity of lipid peroxidation -Enhanced the activity of glutathione, superoxide dismutase, catalase, glutathione peroxidase, glutathione reductase, and glutathione-S-transferase	Glucose tolerance test Oxidative stress enzymatic assay	-Male Wistar rats	-Pancreatic cells -Hepatic cells	[50]
	Glucose Lipid metabolism Inflammatory markers	Oral gavage	1 × 10^9^ colony-forming units/mL	High-fat diet	5 weeks	-Administration of *B. bifidum* significantly reduced plasma glucose level -Treatment with *B. bifidum* increased the adiponectin mRNA level and decreased MCP-1 and IL-6 mRNA levels	Quantitative real-time PCR RNA extraction Glucose tolerance test ELISA Insulin tolerance test	-Swiss-Webster mice -C57BL/6J mice	-Adipose tissue	[52]
*Lactobacillus rhamnosus*	Glucose Lipid metabolism	Orally	1 × 10^9^ colony-forming units/mL	Standard diet	30 days	-Administration of *L. rhamnosus* significantly reduced serum fasting blood glucose -Improved glucose tolerance via downregulation of glucose-6-phosphatase (G6p) expression -Significantly reduced the level of total cholesterol -Lowered the risk of atherosclerosis by lowering the atherogenic index (AI)	Biochemical parameter analysis Glucose tolerance test Quantitative real-time PCR Gene expression analysis	-Sprague-Dawley rats	-Hepatic cells	[56]
	Glucose Inflammatory markers	NA	10^6^ colony-forming units/mL	Fish commercial food	10 days	-Reduced blood glucose level -Supplementation with *L. rhamnosus* resulted in a significant decrease in the expression levels of proinflammatory cytokines -Improved the villus length and width of the intestine	Histological staining Quantitative real-time PCR Immunohistochemistry	-Zebrafish	-Intestinal cells	[58]
	Glucose	Intraperitoneally	1 × 10^9^ colony-forming units/mL	High-fat diet	12 weeks	-Administration of *L. rhamnosus* significantly reduced serum fasting blood glucose -Significantly improved glucose intolerance -Significantly reduced the level of HbA1c and GLP-1	Glucose tolerance test Quantitative real-time PCR Lipid peroxidation inhibiting capacity ELISA Biochemical parameters	-Male C57BL/6J mice		[57]
	Glucose Inflammatory markers	Intraperitoneally Orally	1 × 10^8^ colony-forming units/mL	Chow diet	4 weeks	-Treatment with *L. rhamnosus* significantly improved glucose tolerance -It alleviated endoplasmic reticulum stress by modulating lipid metabolism in skeletal muscle -It alleviated macrophage markers expression F4/80 and CD11b	Glucose tolerance test Real-time PCR Western blot Immunofluorescence	-C57BL/KsJ db/db (db/db) mice	-Adipose tissue -Skeletal muscle	[54]
	Glucose	Intraperitoneally	5 × 10^9^ colony-forming units/mL	High-fat diet	12 weeks	-Treatment with *L. rhamnosus* significantly reduced fasting blood glucose and insulin levels -It significantly decreased glucose-6-phosphatase and phosphoenolpyruvate carboxykinase expression in the livers -It reduced the serum concentrations of proinflammatory cytokines such as tumor necrosis factor alpha (TNFa), interleukin-1b (IL1b), and IL6 -Improved intestinal barrier function in diabetic mice	Glucose tolerance test Histopathological examination Biochemical analysis RNA isolation and RT-PCR analysis Colonic tight junction protein expression analysis	-Male C57BL/6J mice	-Hepatic tissues -Colon tissues	[55]
	Glucose Inflammatory markers	Oral gavage Intraperitoneally	NA	Probiotic fermented milk (PFM)	6 weeks	-PFM significantly improved glucose metabolism (fasting blood glucose, glycated hemoglobin, serum insulin) -It also improved the serum inflammation status (tumor necrosis factor-α, and serum interleukin-6) -PFM has significantly reduced the mRNA expression of pepck and g6pase genes that code the key enzymes of gluconeogenesis pathway	Glucose tolerance test Histopathological examination Biochemical analysis	-Male Wistar rats		[59]
	Glucose Inflammatory markers	Oral gavage Intraperitoneally	NA	High-fat diet	6 weeks	-Treatment with *L. rhamnosus* improved oral glucose tolerance test -It improved the biochemical parameters such as fasting blood glucose, plasma insulin, glycosylated hemoglobin, free fatty acids, triglycerides, total cholesterol, low-density lipoprotein cholesterol, and high-density lipoprotein cholesterol -It also improved the expression of glucagon-like peptide-1-producing genes in the cecum -It reduced the expression of tumor necrosis factor-α and interleukin-6	Glucose tolerance test Biochemical analysis	-Rats		[60]

## Data Availability

Data sharing not applicable.

## Abbreviations

CEs	carbohydrate esterases
CRC	colorectal cancer
DM	diabetes mellitus
FMT	fecal microbiota transplantation
G6pc	glucose-6-phosphatase
GHs	glycosyl hydrolases
GI	gastrointestinal
GLP-1	glucagon-like peptide 1
GLUT	glucose transporter
GTs	glycosyl transferases
HbA1c	hemoglobin A1C (glycated hemoglobin)
HFD	high-fat diet
IL-6	interleukin 6
LDL	light density lipoprotein
NF-B	nuclear Factor kappa-light-chain-enhancer of activated B cells
PFM	probiotic fermented milk
SCFA	short chain fatty acid
STZ	streptozotocin
TC	total cholesterol
TG	triglyceride
TNF	tumor necrosis factor
